# Essential role of the cancer stem/progenitor cell marker nucleostemin for indole-3-carbinol anti-proliferative responsiveness in human breast cancer cells

**DOI:** 10.1186/s12915-014-0072-6

**Published:** 2014-09-12

**Authors:** Antony S Tin, Anna H Park, Shyam N Sundar, Gary L Firestone

**Affiliations:** Department of Molecular and Cell Biology and the Cancer Research Laboratory, 591 LSA, University of California at Berkeley, Berkeley, CA 94720-3200 USA

**Keywords:** nucleostemin, cancer stem/progenitor cell marker, indole-3-carbinol, elastase signaling, nucleostemin–MDM2 interaction, anti-proliferative response in breast cancer cell, tumor xenograft, tumorsphere

## Abstract

**Background:**

Nucleostemin is a GTPase residing in the nucleolus that is considered to be an important cancer stem/progenitor cell marker protein due to its high expression levels in breast cancer stem cells and its role in tumor initiation of human mammary tumor cells. It has been proposed that nucleostemin may represent a valuable therapeutic target for breast cancer; however, to date evidence supporting the cellular mechanism has not been elucidated.

**Results:**

Expression of exogenous HER2, a member of the EGF receptor gene family, in the human MCF-10AT preneoplastic mammary epithelial cell line, formed a new breast cancer cell line, 10AT-Her2, which is highly enriched in cells with stem/progenitor cell-like character. 10AT-Her2 cells display a CD44^+^/CD24^-/low^ phenotype with high levels of the cancer stem/progenitor cell marker proteins nucleostemin, and active aldehyde dehydrogenase-1 (ALDH-1). The overall expression pattern of HER2 protein and the stem/progenitor cell marker proteins in the 10AT-Her2 cell population is similar to that of the luminal HER2+ SKBR3 human breast cancer cell line, whereas both MCF-7 and MDA-MB-231 cells display reduced levels of nucleostemin and no detectable expression of ALDH-1. Importantly, in contrast to the other well-established human breast cancer cell lines, 10AT-Her2 cells efficiently form tumorspheres in suspension cultures and initiate tumor xenograft formation in athymic mice at low cell numbers. Furthermore, 10AT-Her2 cells are highly sensitive to the anti-proliferative apoptotic effects of indole-3-carbinol (I3C), a natural anti-cancer indole carbinol from cruciferous vegetables of the *Brassica* genus such as broccoli and cabbage. I3C promotes the interaction of nucleostemin with MDM2 (murine double mutant 2), an inhibitor of the p53 tumor suppressor, and disrupts the MDM2 interaction with p53. I3C also induced nucleostemin to sequester MDM2 in a nucleolus compartment, thereby freeing p53 to mediate its apoptotic activity. Small interfering RNA knockdown of nucleostemin functionally documented that nucleostemin is required for I3C to trigger its cellular anti-proliferative responses, inhibit tumorsphere formation, and disrupt MDM2–p53 protein–protein interactions. Furthermore, expression of an I3C-resistant form of elastase, the only known target protein for I3C, prevented I3C anti-proliferative responses in cells and in tumor xenografts *in vivo*, as well as disrupting the I3C-stimulated nucleostemin–MDM2 interactions.

**Conclusions:**

Our results provide the first evidence that a natural anti-cancer compound mediates its cellular and *in vivo* tumor anti-proliferative responses by selectively stimulating cellular interactions of the stem/progenitor cell marker nucleostemin with MDM2, which frees p53 to trigger its apoptotic response. Furthermore, our study provides a new mechanistic template that can potentially be exploited for the development of therapeutic strategies targeted at cancer stem/progenitor cells.

## Background

The heterogeneity of human breast cancers results from subpopulations of stem/progenitor cells that possess the capacity for multi-lineage differentiation, and the ability to self-renew and initiate the formation of tumors [1–7]. It has been proposed that the acquired phenotypes of cancer stem cell populations, which constitute approximately 1% to 5% of the cells in primary breast tumors [1], can direct the development of therapy-resistant tumors and relapse of the disease, which significantly influences the effectiveness of a therapeutic strategy [8,9]. Therefore, a critical issue in cancer treatment is the identification of anti-cancer agents that can directly target cancer stem cells to prevent their self-renewal and/or tumor plasticity. However, an experimental constraint that has limited the characterization of stem cell targeted molecules is the low number of cells that can be isolated from stem cell populations enriched *in vitro* in tumorspheres [6,10], or enriched in side-populations of tumor-initiating cells isolated by flow cytometry from primary tumors [6,11,12]. Furthermore, once cultured *in vitro*, the *in vivo* isolated stem cell populations can lose their stem cell character and/or viability.

The orphan epidermal growth factor (EGF) receptor gene family member HER2 (human epidermal growth factor receptor-2) is associated with an enhancement of stem/progenitor cell population levels in populations of either normal mammary epithelial cells or certain cancer cell lines [12,13]. Signaling by HER2 is highly associated with aggressive metastatic forms of breast cancer [14,15], and the gene is amplified in 20% to 30% of all human breast cancers [16]. Expression of exogenous HER2 in normal mammary stem cell populations generated hyperplastic lesions when transplanted *in vivo* [13], and in breast cancer cells HER2 expression enhanced the occurrence of side-populations of tumor-initiating cells of the luminal subtype and is clinically correlated with cancer stem cell populations [12,13,17]. By expressing exogenous HER2 in the MCF-10AT cell line, a well-established model of human mammary epithelial preneoplasia [18], we generated a new breast cancer cell line, denoted as 10AT-Her2, which is highly enriched with cells that display several cancer stem/progenitor cell-like properties. MCF-10AT cells were originally chosen as the starting cell population for our study because of the intrinsic low incidence of tumor formation [18–20] and the lack of any cancer stem cell-like characteristics. In xenografts of immunocompromised mice, a majority of MCF-10AT cells will manifest into normal-appearing ducts; however, a small percentage will displays lesions ranging from atypical hyperplasia to invasive carcinoma [18–20]. It is these qualities that made the parent MCF-10AT cells an ideal candidate system for studying the development of breast cancer via cancer stem/progenitor cells. Cell populations enriched with breast cancer stem cells can be identified by expression of specific sets of marker proteins such as nucleostemin and aldehyde dehydrogenase-1 (ALDH-1), which are associated with maintenance and self-renewal properties [21–24], and by their CD44^+^/CD24^-/low^ phenotype [21]. We observed that the 10AT-Her2 cell population, but not the corresponding 10AT-Neo transfection control cells, express high levels of nucleostemin and active ALDH-1 in the context of a CD44^+^/CD24^-/low^ phenotype, and are able to form tumors xenografts efficiently *in vivo* in athymic mice and form tumorspheres in suspension cultures at limiting cell dilutions. The 10AT-Her2 cell system provided the experimental opportunity directly to test the hypothesis that cellular components that help define the cancer stem/progenitor character, such as nucleostemin, can confer selective responsiveness of anti-cancer compounds to target breast cancer stem/progenitor populations.

Indole-3-carbinol (I3C), a natural indole carbinol produced from the hydrolysis of glucobrassicinin, is found in cruciferous vegetables of the *Brassica* genus, such as broccoli and cabbage, and is a promising anti-cancer compound [25–29]. I3C treatment activates distinct sets of anti-proliferative signaling cascades in a wide range of human breast cancer cells [25,30–40], inhibits the *in vivo* growth of human breast cancer cell-derived tumor xenografts [34], and reduces tumor metastasis and breast cancer cell migration [35,41]. Clinical trials have concluded that ingested I3C possesses anti-cancer effects in human populations, has beneficial effects on estrogen metabolism [42], and, based on cytotoxicity studies, patients can receive as high as 800 mg/kg/day of I3C without any adverse side effects [43–45]. It is important to note that the functional intracellular concentration of I3C is significantly lower than the treated amount in *in vitro* and *in vivo* studies because in cell culture studies only approximately 0.3% of extracellular I3C enters a cell [46].

A key advance in understanding the molecular mechanism of the anti-cancer actions of indole carbinols is our discovery that I3C and its highly potent derivative 1-benzyl-I3C [47], but not the natural I3C dimerization product 3,3'-dimethylindolylmethane (DIM), act as direct noncompetitive inhibitors of elastase enzymatic activity, the first such identified target protein for I3C [33,34,48]. Intriguingly, a high level of elastase activity has been associated with late stage breast cancer [49]. *In silico* simulations that model I3C interactions with the crystallographic structure of elastase, uncovered a critical interaction site for I3C (and 1-benzyl-I3C) that provided the experimental foundation for generating a truncated form of elastase that is enzymatically active but resistant to inhibition by either I3C or 1-benzyl-I3C [48]. Using this unique reagent, we demonstrate that I3C triggers an elastase-dependent anti-proliferative response in the 10AT-Her2 breast cancer cell population by promoting nucleostemin to interact with and sequester the murine double mutant 2 (MDM2) protein into the nucleolus, thereby allowing the p53 tumor suppressor protein to escape from the MDM2 inhibition of apoptotic activity. Our study has uncovered new mechanistic insights into how the cancer stem/progenitor cell-associated component nucleostemin is directly involved in an anti-proliferative cell signaling pathway triggered by I3C, a natural anti-cancer molecule.

## Results

### Expression of exogenous HER2 in preneoplastic mammary epithelial cells induces a stable cancer stem/progenitor cell-like phenotype

**Figure 1:**
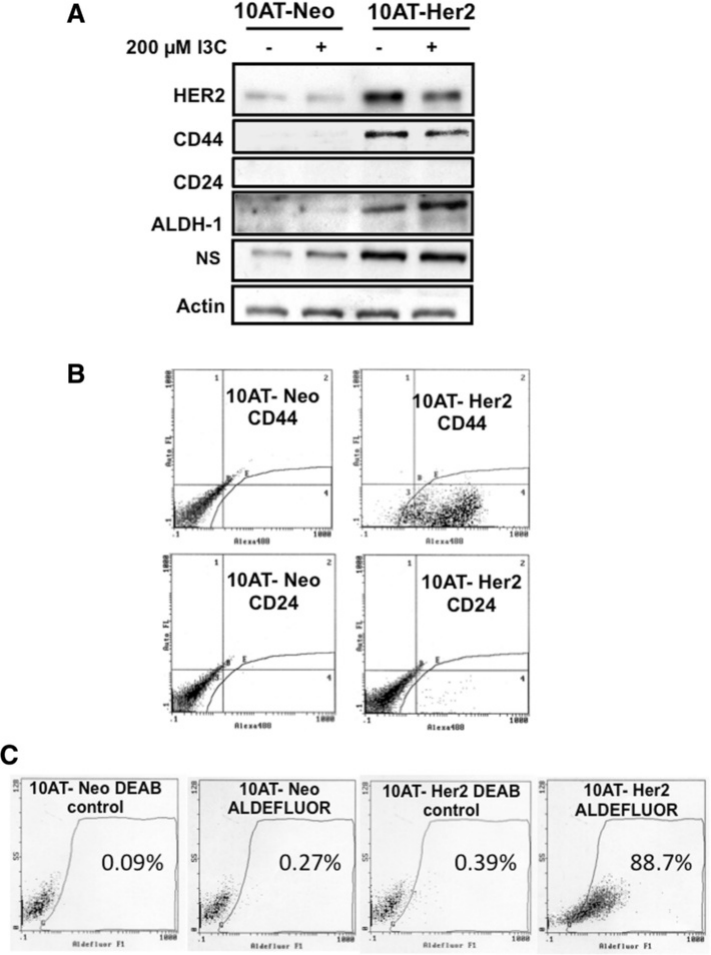
**Expression of cancer stem/progenitor cell-like marker proteins in 10AT-Her2 and 10AT-Neo cells. (A)** Cultured HER2-expressing 10AT-Her2 cells and empty vector transfected 10AT-Neo control cells were treated with or without 200 μM I3C for 48 hours. Production of HER2, CD44, CD24, ALDH-1, nucleostemin (NS) and actin protein were determined by Western blot analysis of electrophoretically fractionated total cell extracts. **(B)** Cell surface expression of CD44 and CD24 in 10AT-Her2 cells and 10AT-Neo cells was quantified by flow cytometry of 500,000 cells in triplicate independent cell cultures. **(C)** ALDH-1 activity was quantified in 10AT-Her2 and 10AT-Neo cells by ALDEFLUOR assay as described in the Methods section. ALDH-1, aldehyde dehydrogenase-1; HER2, human epidermal growth factor receptor-2; NS, nucleostemin.

To generate a mammary epithelial cancer cell system highly enriched with tumor-initiating cells that express high levels of nucleostemin, preneoplastic MCF-10AT human mammary epithelial cells [18–20] were stably transfected with either the CMV-HER2 expression vector containing the neomycin resistance gene, or the control CMV-neomycin resistance gene vector forming 10AT-Her2 and 10AT-Neo cells, respectively. Western blot analysis demonstrated that 10AT-Her2 cells expressed significantly higher levels of HER2 compared to the control 10AT-Neo cell line (Figure 1A, top panel). I3C-treated conditions will be discussed in a later section. Western blots also demonstrated that the 10AT-Her2 cell population is highly enriched with cells that express significantly elevated levels of nucleostemin, ALDH-1 and CD44, and the maintenance of nearly undetectable levels of CD24 (Figure 1A), which is a phenotype associated with a cancer stem cell/progenitor cell-like character. In contrast, the control 10AT-Neo cell population maintained the same phenotype as the starting preneoplastic MCF-10AT cells with nearly undetectable to low levels of CD44, CD24, ALDH-1 and nucleostemin.

To determine the percentage of cells within the 10AT-Her2 cell population that display stem/progenitor cell-like marker proteins, the levels of cell surface-associated CD44 and CD24 were quantified by flow cytometry using antibodies specific for either CD44 or CD24. As shown in Figure 1B, greater than 98% of 10AT-Her2 cells in the population express high levels of CD44 compared to the background levels observed with control transfected 10AT-Neo cells. The level of CD24 remained low in both cell lines and was expressed in less than 2% of the cell population (Figure 1B). An ALDEFLUOR assay verified that greater than 88% of the ALDH-1 in the 10AT-Her2 cell population is enzymatically active while less than 1% of the ALDH-1 in 10AT-Neo cells was active (Figure 1C). The elevated level of ALDH-1 activity in the 10AT-Her2 cell population is consistent with previous studies showing that the subpopulations within normal and cancer human mammary epithelial cells with increased ALDH-1 have stem/progenitor cell-like properties [24]. The phenotype of the overall 10AT-Her2 cell population, which displays high levels of nucleostemin and active ALDH-1 in a CD44^+^/CD24^–/low^ phenotype background, has remained stable after continuous culturing of this cell line for more than 6 months over many cell generations.

**Figure 2:**
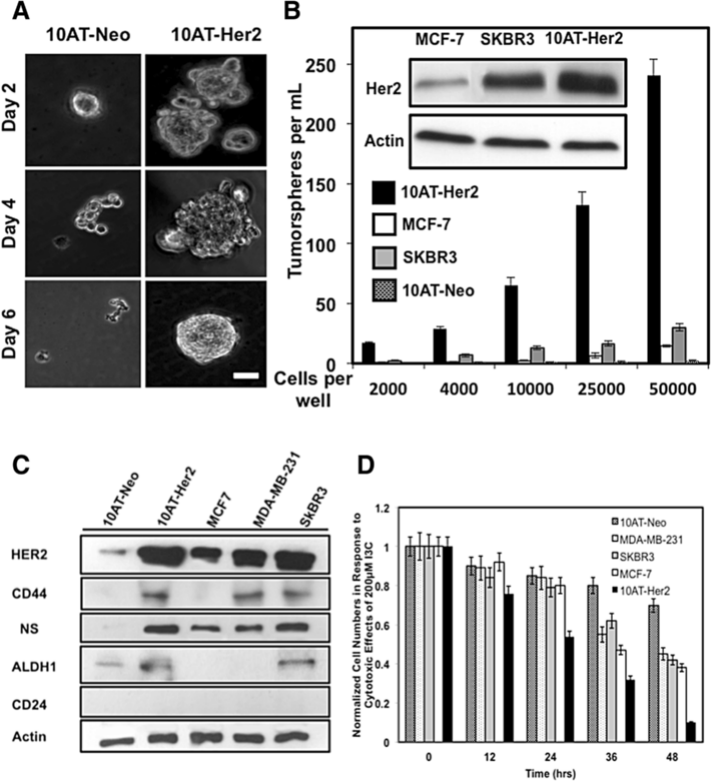
**Tumorsphere formation efficiency in cell suspension cultures, expression of stem/progenitor cell marker proteins and proliferation of breast cancer cells. (A)** 10AT-Her2 and 10AT-Neo cells were plated at a density of 4,000 cells per well in serum-free non-adherent suspension cultures as described in the Methods section. At the indicated days in culture, tumorsphere formation was assessed visually by phase microscopy. Scale bar represents 50 μm. **(B)** 10AT-Her2 and 10AT-Neo cells as well as the SKBR3 and MCF-7 human breast cancer cell lines were incubated at the indicated cell densities. Tumorsphere formation efficiency was quantified after 6 days in culture under non-adherent conditions. The presented values are an average of three independent experiments. The gel inserts are Western blots showing relative levels of HER2 protein expression and actin controls from electrophoretically fractionated total cell extracts of MCF-7, SKBR3 and 10AT-Her2 cells. **(C)** Cultured 10AT-Neo, 10AT-Her2, MCF-7, MDA-MB-231 and SKBR3 cells were harvested, total cell extracts electrophoretically fractionated and the levels of expressed HER2, CD44, CD24, ALDH-1, nucleostemin (NS) and actin protein determined by Western blots. **(D)** To examine the effects of I3C on cell proliferation, 10AT-Neo, 10AT-Her2, SKBR3, MCF-7 and MDA-MB-231 cells cultured in a 24-well plate were treated with 200 μM I3C for the indicated times and the cell number quantified as described in the Methods section. ALDH-1, aldehyde dehydrogenase-1; HER2, human epidermal growth factor receptor-2; NS, nucleostemin.

The *in vitro* formation of tumorspheres in cell suspension cultures is considered a cellular property of cancer stem/progenitor cells within a cell population that is predictive of tumor initiation properties [6,23]. 10AT-Her2 and 10AT-Neo cells were therefore cultured at low density (approximately 4,000 cells/ml) in cell suspensions, and *in vitro* tumorsphere formation was monitored visually for 6 days. As shown in Figure 2A, 10AT-Her2 cells began to form tumorsphere-like structures within 2 days of culture and by 6 days the cells formed completed tumorspheres. In contrast, the control 10AT-Neo cells failed to form tumorspheres and remained dispersed in small cell aggregates. The tumorsphere-forming efficiency of 10AT-Her2 cells was compared to that of two luminal subtype tumorigenic breast cancer cell lines, SKBR3 and MCF-7, which differ in their expression of HER2 [50]. The Western blot insert in Figure 2B shows that SKBR3 and 10AT-Her2 cells produce approximately the same levels of HER2 protein, whereas MCF-7 cells produce significantly lower levels of HER2. By culturing increasing numbers of cells in suspension, the 10AT-Her2 cells were more than tenfold more efficient in their tumorsphere-forming capability compared to either SKBR3 or MCF-7 breast cancer cells (Figure 2B). For example, the number of tumorspheres formed from 2,000 10AT-Her2 cells was observed only when 25,000 SKBR3 cells or 50,000 MCF-7 cells were assayed. Also, because SKBR3 and 10AT-Her2 express similar levels of HER2 protein, the ability of 10AT-Her2 cells to form tumorspheres cannot be attributed only to the high level of exogenous HER2.

To compare the expression of cancer stem/progenitor cell-like marker protein in the 10AT-Her2 cell population with other well-established human breast cancer cell lines, protein levels of nucleostemin, CD44, ALDH-1 and CD24 were assessed in 10AT-Neo, 10AT-Her2, MCF-7, MDA-MB-231 and SKBR3 cells (Figure 2C). As mentioned above, MCF-7 and SKBR3 cells represent two distinct luminal subtypes, whereas, MDA-MB-231 cells represent a triple negative basal subtype. Western blot analysis indicated that 10AT-Her2 cells and SKBR3 cells express relatively comparable levels of HER2, CD44, nucleostemin and ALDH-1 protein, although the tumorsphere-forming properties of SKBR3 cells are significantly less efficient than that of 10AT-Her2 cells (Figure 2B). Densitometric analysis of the Western blots show that both MCF-7 cells and MDA-MB-231 breast cancer cells express approximately 30% of the levels of nucleostemin compared to either 10AT-Her2 or SKBR3 cells. The production of ALDH-1 was not detected in either MCF-7 cells or MDA-MB-231 cells, whereas, MCF-7 cells also do not express CD44. These results show that even though well-established breast cancer cell lines express specific stem/progenitor cell-like protein markers, the 10AT-Her2 cell population can be considered to have a more enhanced ‘stemness’ character because of its highly efficient tumorsphere-formation property (Figure 2B), and as discussed in later sections, its ability to form tumor xenografts at low cell numbers in athymic mice.

The anti-proliferative effects of I3C were analyzed in 10AT-Her2 cells and 10AT-Neo cells in comparison to MCF-7, MDA-MB-231 and SKBR3 breast cancer cells, which we have previously shown to be sensitive to this natural indole carbinol compound [31–34,38–41]. Cells were treated with 200 μM I3C, which is the optimal concentration for its anti-cancer effects in breast cancer cell lines [32–34,39,41], and the number of cells in each cell culture well were quantified throughout 48 hours. As shown in Figure 2D, of the tested cell lines, 10AT-Her2 cells were the most sensitive to the cytotoxic effects of I3C and displayed a near complete loss of cell proliferation after 48 hours of treatment with I3C. In contrast, the transfection control 10AT-Neo cells were only mildly sensitive to the cytotoxic effects of I3C. Each of the three well-established breast cancer cell lines show similar levels of sensitivity to I3C, although the effect was not as efficient as observed in the 10AT-Her2 cells.

### I3C disrupts *in vitro* 10AT-Her2 cell tumorsphere formation and *in vivo* tumor xenograft growth

**Figure 3:**
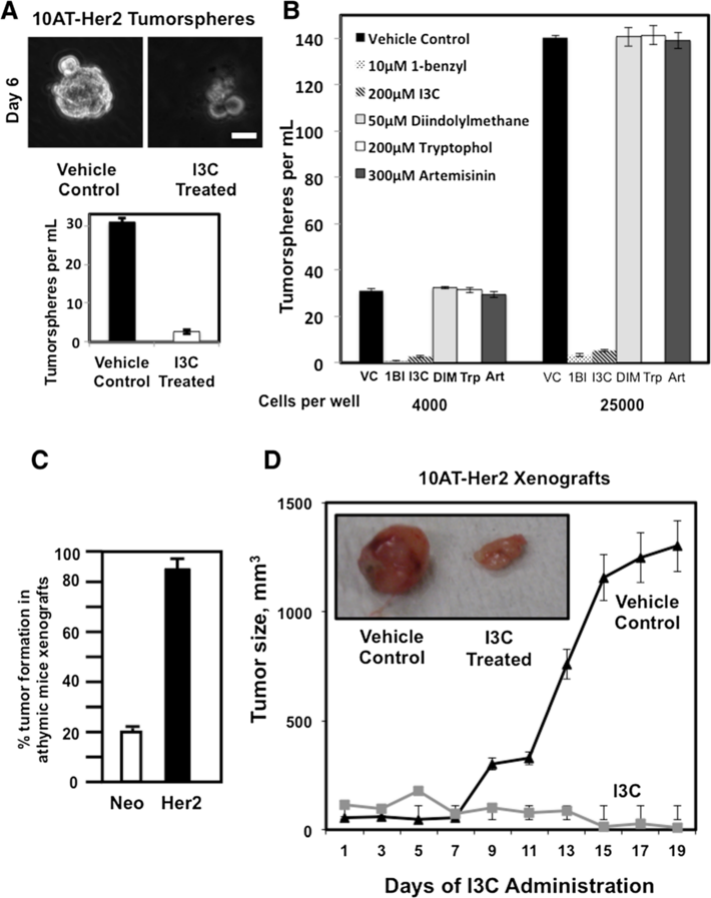
**Tumor xenograft-forming efficiency and tumorsphere and** ***in vivo*** **growth inhibition by I3C. (A)** 10AT-Her2 cells were plated at a density of 4,000 cells per well in tumorsphere culture conditions and incubated with or without 200 μM I3C. After 6 days in cell suspension cultures, tumorsphere formation was assessed visually by phase microscopy and quantified. Scale bar represents 50 μm. **(B)** 10AT-Her2 cells were plated at a density of either 4,000 cells per well or 25,000 cells per well in tumorsphere culture conditions and then incubated with 10 μM 1-benzyl-I3C (1BI), 200 μM I3C, 50 μM DIM, 200 μM tryptophol (Trp), 300 μM artemisinin (Art) or with the DMSO vehicle control (VC). After 6 days in cell suspension cultures, tumorsphere formation was assessed visually by phase microscopy and quantified. **(C)** Three million 10AT-Neo cells (Neo) or 300,000 10AT-Her2 cells (Her2) were implanted in athymic mice, and 20 separate tumor injection sites were examined for palpable tumors after 5 weeks and quantified. **(D)** After the formation of detectable 10AT-Her2 cell-derived palpable tumor xenografts, athymic mice were treated subcutaneously with either 300 mg/kg of I3C or the DMSO vehicle control. Tumor volumes were quantified with a caliper from ten tumor xenografts per condition using two tumor sites per animal. The micrograph insert shows representative tumors excised from 2-week-post injection animals. 1BI, 1-benzyl-I3C; Art, artemisinin; DIM, 3,3'-dimethylindolylmethane; Her2, 10AT-Her2; I3C, indole-3-carbinol; Neo, 10AT-Neo; Trp, tryptophol; VC, vehicle control.

The potential I3C inhibition of the *in vitro* formation of 10AT-Her2 cell tumorspheres was examined in cell suspensions that were treated for 6 days with or without 200 μM I3C. Analysis by light microscopy revealed that I3C completely prevented the *in vitro* formation of tumorspheres (Figure 3A). Only approximately 0.3% of I3C enters breast cancer cells from the cell culture medium [46], so the functional intracellular concentration of this indole carbinol compound is significantly lower than that added to the cell cultures. Quantification of the efficiency of 10AT-Her2 cell tumorsphere formation demonstrated that I3C had strong inhibitory effects on this cancer stem/progenitor cell-like process (Figure 3A, bar graphs). This inhibitory effect on *in vitro* 10AT-Her2 cell tumorsphere formation was specific for I3C-based indole carbinol compounds because the highly potent I3C derivative 1-benzyl-I3C [47] inhibited tumorsphere formation by 98% at significantly lower concentrations than I3C; whereas, the inactive indole carbinol compound tryptophol, which is structurally similar to I3C [47], had no effect on tumorsphere formation (Figure 3B). Other phytochemicals that display strong anti-proliferative responses in a variety of breast cancer cell lines, such as the natural I3C dimer, DIM [28], and artemisinin [51], had no effect on 10AT-Her2 cell tumorsphere formation (Figure 3B). Therefore, the disruption of tumorsphere formation in cell suspension cultures is a property specific to I3C and its highly potent derivative compared to other phytochemicals that can target breast cancer cells.

The *in vivo* tumor-initiating capability of the 10AT-Her2 cell line in comparison to control 10AT-Neo cells was analyzed by formation of tumor xenografts in NIH III athymic nude mice. When 3 million control 10AT-Neo cells were injected into the athymic mice, this cell line displayed a 20% to 25% tumor efficiency, which is consistent with sporadic events associated with the preneoplastic nature of the parental MCF-10AT cell line (Figure 3C). In contrast, 10AT-Her2 cells were highly efficient in the ability to form tumor xenografts in athymic mice. In a series of limiting dilution studies, injection of 300,000 10AT-Her2 cells form tumor xenografts at nearly 100% efficiency (Figure 3C) and are capable of tumor initiation when injections are carried out with as few as 20,000 cells (data not shown). The ability of 10AT-Her2 cells to form tumor xenografts *in vivo* is tenfold more efficient than that reported for highly tumorigenic human breast cancer cell lines such as MCF-7, MDA-MB-231 and SKBR3, which require approximately 2 million cells for tumor xenografts to be observed [52].

To assess the *in vivo* effects of I3C on the growth of 10AT-Her2-cell-derived tumor xenografts, 300,000 10AT-Her2 cells were injected into NIH III athymic mice and the resulting tumors were first allowed to grow to an average volume of approximately 100 mm^3^. The mice were then injected subcutaneously with either I3C (300 mg/kg body mass) or with the dimethyl sulfoxide (DMSO) vehicle control over 19 days. In vehicle-control-treated animals, the 10AT-Her2 cell tumor xenografts showed robust growth (Figure 3D). The concentration of I3C used for the mice injections is approximately equivalent to the 200 μM I3C used to treat the cultured cell lines. Also, in phase 1 clinical trials, women have been given as high a dose as 800 mg I3C per day with high tolerability [43–45], suggesting that relatively high doses of I3C can be tolerated without any adverse side effects. In the absence of I3C, the resulting tumor xenografts displayed highly concentrated gross tumor vascularization and were dense (Figure 3D, micrograph insert), consistent with the rapid growth of cells within the tumor. I3C strongly suppressed the growth of 10AT-Her2 cell-derived tumor xenografts (Figure 3D), and the resulting tumors appeared less vascularized and much smaller in size (Figure 3D, micrograph insert). The texture of the residual tumors from I3C-treated mice was pliable, consistent with reduced cell density in the xenografts. Coupled with the fact that 10AT-Her2 cells are ten times more efficient at forming tumorspheres *in vitro* (Figure 2B), our *in vivo* results strongly suggest that the 10AT-Her2 cell population is highly enriched with cancer stem/progenitor-like cells with an efficient tumor-initiation capability.

### I3C induces a p53-dependent apoptotic response and promotes the interaction of the stem cell marker protein nucleostemin with the MDM2 in 10AT-Her2 cells

**Figure 4:**
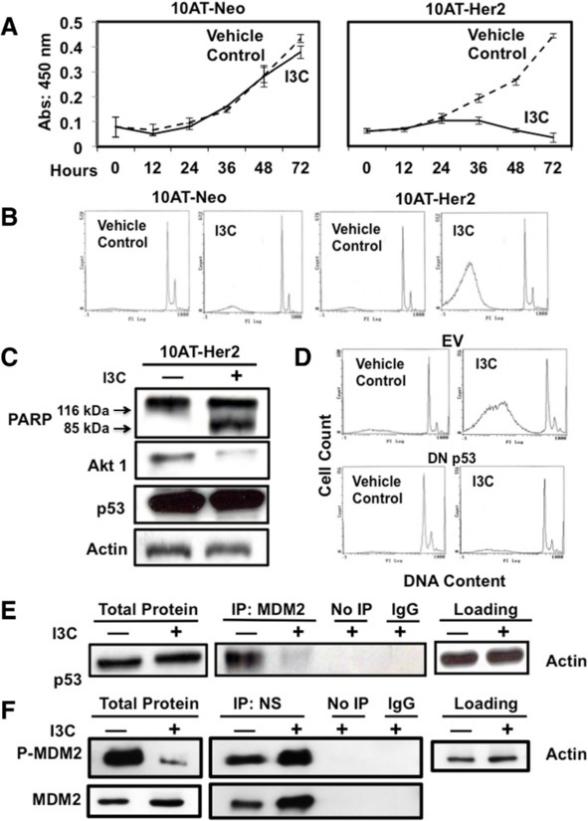
**I3C induces p53-dependent apoptosis and regulation of MDM2–p53 and MDM2–nucleostemin protein–protein interactions. (A)** 10AT-Her2 and 10AT-Neo cells were treated with or without 200 μM I3C for the indicated durations and cell number was quantified by the cell proliferation assay described in the Methods section. **(B)** 10AT-Neo and 10AT-Her2 cells were treated with or without 200 μM I3C for 48 hours. The DNA content of nuclear DNA stained with propidium iodide was assessed by flow cytometry. **(C)** 10AT-Her2 cells were treated with or without 200 μM I3C for 48 hours, total cell extracts were electrophoretically fractionated and then Western blots probed for PARP, Akt1, p53 and the actin gel loading control. **(D)** 10AT-Her2 cells were transfected with either a dominant negative p53 (DN p53) expression vector or with the empty expression vector (EV), and then treated with or without 200 μM I3C for 48 hours. The DNA content of nuclear DNA stained with propidium iodide was assessed by flow cytometry. **(E, F)** 10AT-Her2 cells were treated with or without 200 μM I3C for 48 hours. Total cell extracts were immunoprecipitated with either MDM2 **(E)** or nucleostemin **(F)** antibodies. As a control, non-immune antibodies (of immunoglobulin G or IgG) and samples not immunoprecipitated (No IP) were used. All extracts were electrophoretically fractionated and probed by Western blot analysis using antibodies specific to p53, serine-166 phosphorylated MDM2 or total MDM2 **(E)** or with antibodies specific to either serine-166 phosphorylated MDM2 or total MDM2 **(F)**. DN, dominant negative; EV, empty expression vector; I3C, indole-3-carbinol; IgG, immunoglobulin G; IP, immunoprecipitated; MDM2, murine double mutant 2; PARP, poly ADP ribose polymerase.

To assess the anti-proliferative and apoptotic effects of I3C, 10AT-Her2 cells and 10AT-Neo cells were initially cultured in adherent monolayers and treated with or without 200 μM I3C over 72 hours. The total cell number was determined using the cell-counting kit-8 assay [53]. I3C rapidly prevented the proliferation of 10AT-Her2 cells with a maximal response observed by 72 hours (Figure 4A, right panel), whereas, proliferation of the control 10AT-Neo cells remained relatively unaffected by I3C treatment (Figure 4A, left panel). Flow cytometry of nuclear DNA stained with propidium iodide revealed that 48 hours of I3C treatment induced a significant increase in 10AT-Her2 cells with a sub-G1 DNA content compared to vehicle-control-treated cells, which is indicative of the activation of apoptosis (Figure 4B, right panels). The control 10AT-Neo cells remained relatively resistant to I3C and displayed only a very minor increase in cells with a sub G1-DNA content (Figure 4B, left panels). Under these conditions, I3C treatment of 10AT-Her2 cells did not alter the expression of the cancer stem/progenitor cell-like marker proteins nucleostemin, CD44, CD24 and ALDH-1 (Figure 1A).

To determine whether I3C induced an apoptotic response and verify the sub-G1 DNA content observed by flow cytometry, 10AT-Her2 cells were treated with or without I3C. Western blots probed for the production of cleaved poly ADP ribose polymerase (PARP) protein, a substrate of activated caspase 3 in the apoptotic pathway [54]. As shown in Figure 4C, I3C treatment strongly stimulated PARP cleavage as shown by the detection of significant levels of the 85-kDa PARP cleavage product, and 10AT-Her2 cells express similar levels of the p53 tumor suppressor protein in the presence or absence of I3C. It is well established that regulation of the p53 tumor suppressor protein plays an important role in the control of cellular apoptosis [55]. Therefore, the potential role of p53 in the I3C apoptotic response was functionally evaluated by transfection of 10AT-Her2 cells with either a dominant negative (DN) p53 or an empty expression vector (EV) as a control. Flow cytometry of nuclear DNA stained with propidium iodide from 48-hour I3C-treated or untreated cells revealed that expression of DN p53 prevented the I3C-stimulated production of sub-G1 DNA content, which is indicative of loss of apoptosis, whereas I3C efficiently induced an apoptotic response in 10AT-Her2 cells transfected with the empty vector (Figure 4D).

One known mechanism by which the p53-dependent apoptotic response can be regulated is through the direct binding of MDM2 to p53, which prevents the apoptotic activity of p53 by sequestering p53 away from its apoptotic targets [56]. MDM2 co-immunoprecipitations were carried out to determine whether I3C treatment disrupts the ability of MDM2 to interact with p53. 10AT-Her2 cells were treated with or without I3C for 48 hours and the immunoabsorbed MDM2 protein complex was electrophoretically fractionated and Western blots probed for the presence of p53 in the immunoabsorbed protein. In the absence of I3C, a significant amount of p53 protein co-immunoprecipitated with MDM2, which shows the presence of the MDM2–p53 protein interaction in proliferating cells, whereas, in contrast, after I3C treatment, the MDM2–p53 protein interaction is nearly ablated (Figure 4E). This result suggests that the I3C disruption of MDM2 binding to p53 frees this tumor suppressor protein to trigger its apoptotic response. Consistent with this concept, expression of a constitutively active form of Akt-1, which phosphorylates MDM2 and promotes MDM2 binding to p53 [56], prevented the I3C apoptotic response and restored MDM2–p53 protein interactions (data not shown).

Because I3C triggers anti-proliferative signaling in 10AT-Her2 cells through a p53- dependent response, we examined whether cellular components that define the cancer stem/progenitor cell-like phenotype may be associated with the I3C regulation of the MDM2–p53 pathway. One such intriguing molecular marker that is highly expressed in self-renewing cancer stem/progenitor cells and is associated with the MDM2–p53 pathway is nucleostemin [57–59], which is a nuclear GTPase that has been shown to interact directly with MDM2 [57,60,61]. There is only limited information concerning the regulation or functional significance of nucleostemin–MDM2 protein interactions in human cancer cells [60,61]. I3C had no effect on the total levels of nucleostemin protein (see Figure 1A) or total MDM2 (Figure 4F, left panel) expressed in 10AT-Her2 cells, although the level of detectable serine-166 (Ser166) phosphorylated MDM2 decreased in I3C-treated cells (Figure 4F, left panel). Co-immunoprecipitations were carried out by immunoadsorbing nucleostemin from 48-hour I3C-treated or untreated cells and then Western blots probed for either the Ser166 phosphorylated MDM2 or total MDM2 protein. As shown in Figure 4F, I3C treatment strongly enhanced nucleostemin interactions with both the Ser166 phosphorylated MDM2 protein and the total MDM2 protein. This result suggests that the I3C-induced interaction of nucleostemin with the Ser166 phosphorylated form of MDM2 prevents p53 from binding to MDM2 and accounts for the ability of this natural indole carbinol compound to trigger a p53-dependent apoptotic response in 10AT-Her2 cells.

**Figure 5:**
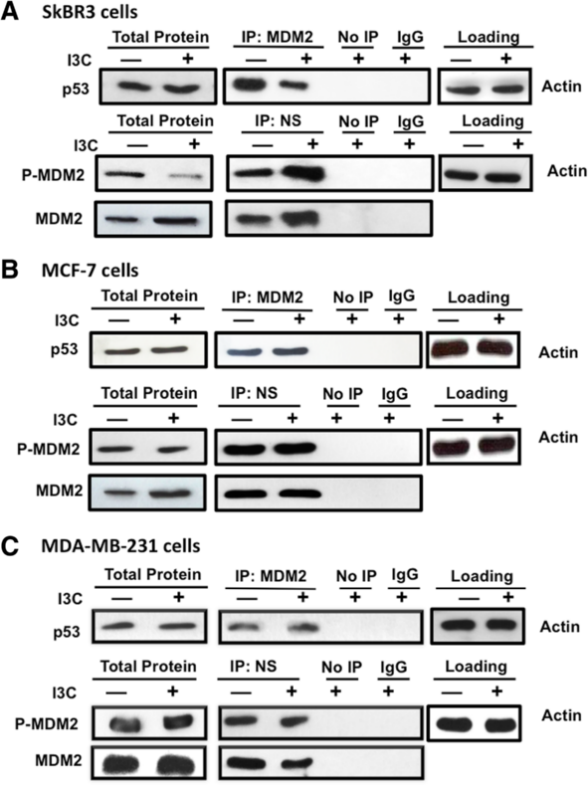
**I3C regulation of nucleostemin–MDM2 and p53-MDM2 protein interactions in well-established human breast cancer cell lines.** SKBR3 **(A)**, MCF-7 **(B)** and MDA-MB-231 **(C)** human breast cancer cells were treated with or without 200 μM I3C for 48 hours. Total cell extracts were immunoprecipitated with either MDM2 (top panels for each cell line) or nucleostemin (lower panels for each line) antibodies. As a control, non-immune antibodies (IgG) and samples not immunoprecipitated (No IP) were used. All extracts were electrophoretically fractionated and probed by Western blot analysis using antibodies specific to p53 (top panels) or with antibodies specific to serine-166 phosphorylated MDM2 or total MDM2 (lower panels). The levels of actin protein remaining in the cell extracts after the immunoprecipitations were used as gel-loading controls in each experiment. I3C, indole-3-carbinol; IgG, immunoglobulin G; IP, immunoprecipitated; MDM2, murine double mutant 2; NS, nucleostemin.

To assess whether the I3C regulation of MDM2 protein interactions with p53 and/or nucleostemin occurs in other indole-carbinol-sensitive breast cancer cells, three well-established cell lines, SKBR3, MCF-7 and MDA-MB-231, were treated with or without I3C for 48 hours and MDM2–p53 and nucleostemin–MDM2 co-immunoprecipitations carried out as described above for 10AT-Her2 cells. As shown in Figure 5A, I3C disrupted MDM2–p53 interactions and stimulated nucleostemin–MDM2 interactions in SKBR3 cells, a cell line that expresses nucleostemin and other stem/progenitor cell-like marker proteins approximately to the same levels as the 10AT-Her2 cell population (Figure 2C). Therefore, the effects of I3C on nucleostemin–MDM2 and MDM2–p53 interactions that we observed with 10AT-Her2 cells is not limited only to this newly developed breast cancer cell line. In contrast, even though MCF-7 and MDA-MB-231 cells are sensitive to the anti-proliferative effects of I3C, there were no detectable changes in MDM2–p52 or nucleostemin–p53 protein interactions after I3C treatment (Figure 5B,C). Based on expression of marker proteins, the relative stemness character of the MCF-7 and MDA-MB-231 cell populations can be considered less than that of either SKBR3 or 10AT-Her2 cells, which may be associated with the lack of any effects of I3C treatment on nucleostemin protein–protein interactions.

### Interfering RNA knockdown of nucleostemin in 10AT-Her2 cells disrupts the I3C-stimulated localization of MDM2 into the nucleolus compartment, strongly attenuates the I3C-induced apoptotic response and partially reverses the loss of MDM2–p53 interactions

**Figure 6:**
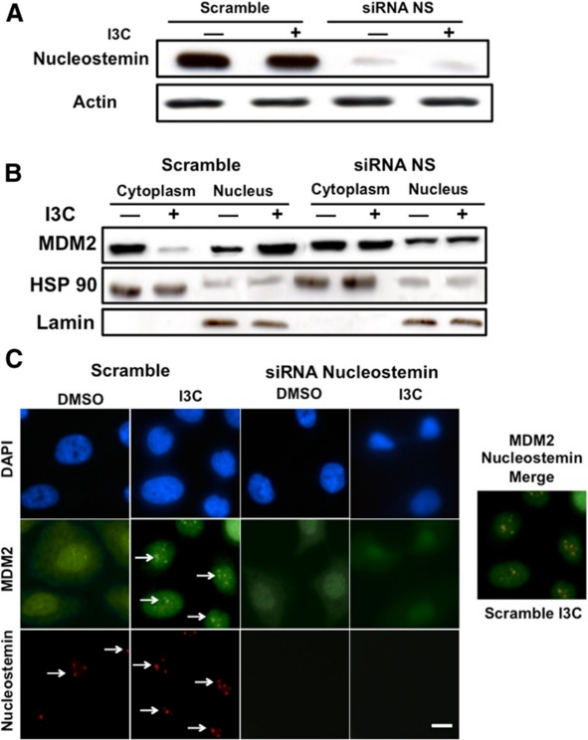
**Nucleostemin-dependent I3C stimulation of MDM2 nuclear compartmentalization and localization into nucleolus foci.** 10AT-Her2 cells were transfected with either control scramble siRNA or nucleostemin siRNA, and then treated with or without 200 μM I3C for 48 hours. **(A)** The level of nucleostemin protein was determined by Western blot analysis. **(B)** Cell extracts were biochemically separated into nuclear enriched and cytoplasmic fractions, electrophoretically fractionated, and Western blots probed with antibodies specific for MDM2, the cytoplasmic marker HSP90 and the nuclear marker lamin. **(C)** The subcellular localization of MDM2 and nucleostemin was determined by indirect immunofluorescence microscopy. DAPI staining was used to visualize DNA stained nuclei. Scale bar represents 4 μm. DAPI, 4',6-diamidino-2-phenylindole; DMSO, dimethyl sulfoxide; I3C, indole-3-carbinol; MDM2, murine double mutant 2; NS, nucleostemin; siRNA, small interfering RNA.

Given that nucleostemin resides in the nucleolus [57] and MDM2 translocates between the cytoplasm and nucleus [56], an intriguing issue is whether the I3C-induced nucleostemin- MDM2 interaction drives the localization of MDM2 to the nucleus in I3C-treated cells. This possibility was functionally examined by siRNA knockdown of nucleostemin. Western blots showed that nucleostemin siRNA efficiently reduced the levels of nucleostemin compared to cells receiving scrambled siRNA (Figure 6A). The localization of MDM2 was initially examined in cells treated for 48 hours with or without I3C and then biochemically fractionated into nuclear and cytoplasmic extracts. Western blots showed that in cells transfected with scrambled siRNA, I3C treatment causes the redistribution of MDM2 from cytoplasmic and nuclear fractions into predominantly the nuclear fraction (Figure 6B). In contrast, knockdown of nucleostemin prevented the I3C-induced subcellular localization of MDM2 into the nuclear fraction. Under each condition, the cytoplasmic fraction remained enriched in the cytoplasmic marker HSP90, whereas the nuclear compartment was enriched in nuclear marker lamin.

In 10AT-Her2 cells transfected with scrambled siRNA, indirect immunofluorescence revealed that nucleostemin is localized to punctate foci in the nucleus in the presence or absence of I3C, which is indicative of the nucleolus compartment (Figure 6C, left set of panels). Strikingly, I3C treatment triggered the redistribution of MDM2 from both the nucleus and cytoplasm, to an enriched punctate staining pattern as foci in the nucleus (Figure 6C, left set of panels, see arrows). Merging of the nucleostemin and MDM2 immunofluorescence staining in the scrambled siRNA transfected cells treated with I3C revealed that nucleostemin and MDM2 co-localize into identical foci staining patterns (Figure 6, merged staining panel). Importantly, siRNA knockdown of nucleostemin completely disrupted the nuclear foci staining of MDM2 in I3C-treated cells, and the overall localization of MDM2 resembled that observed in cells not treated with I3C (Figure 6C, right set of panels).

**Figure 7:**
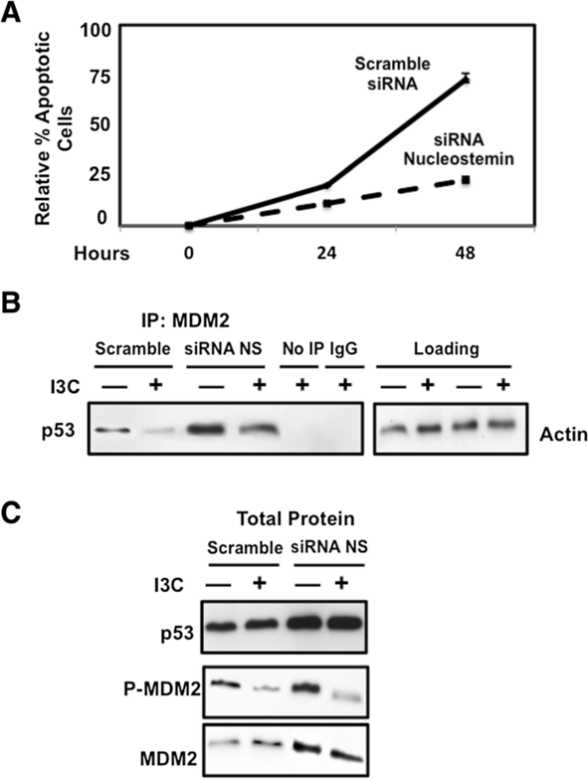
**Effects of nucleostemin knockdown on I3C-induced apoptotic response and regulated MDM2- p53 protein interactions. (A)** 10AT-Her2 cells transfected with nucleostemin siRNA or with scrambled siRNA control were treated with or without 200 μM I3C for the indicated durations. The relative amount of apoptosis was quantified by the ratio of sub-G1 DNA content as determined by flow cytometry. The number of apoptotic cells observed after I3C treatment was normalized to the number of apoptotic cells observed in DMSO-vehicle-control-treated cells. **(B)** 10AT-Her2 cells were transfected with either scrambled siRNA or nucleostemin-specific siRNA, and then treated with or without 200 μM I3C for 48 hours. Total cell extracts were immunoprecipitated with MDM2 antibodies, electrophoretically fractionated and Western blots probed with p53-specific antibodies. Negative control immunoprecipitations were carried out with non-immune antibodies (IgG) or samples that were not immunoprecipitated (No IP). **(C)** Western blots of total cell extracts (total protein) were analyzed for p53, serine-166 phosphorylated MDM2 and total MDM2. DMSO, dimethyl sulfoxide; I3C, indole-3-carbinol; IgG, immunoglobulin G; IP, immunoprecipitated; MDM2, murine double mutant 2; NS, nucleostemin; siRNA, small interfering RNA.

To determine whether the I3C apoptotic response in 10AT-Her2 cells requires expression of nucleostemin, cells transfected with either nucleostemin or scrambled siRNA were treated with or without I3C for 48 hours. The relative apoptotic response was quantified as the ratio of sub-G1 content DNA in untreated to I3C-treated cells. As shown in Figure 7A, siRNA knockdown of nucleostemin significantly attenuated the apoptotic response compared to that observed in 10AT-Her2 cells transfected with scrambled siRNA. Co-immunoprecipitations of I3C-treated and untreated cells demonstrated that knockdown of nucleostemin partially reversed the I3C disruption of MDM2–p53 protein interactions (Figure 7B). The level of MDM2–p53 interactions in I3C-treated cells transfected with nucleostemin siRNA was approximately the same as vehicle-control-treated scramble siRNA transfected cells (Figure 7B). Interestingly, siRNA knockdown of nucleostemin increased the overall levels of p53. Also, I3C treatment efficiently inhibited production of Ser166 phosphorylated MDM2 regardless of the presence of nucleostemin (Figure 7C), suggesting that this regulated step is upstream of the I3C-regulated process that affects nucleostemin. Taken together, these results demonstrate that I3C requires the nucleostemin cancer stem cell marker to trigger its apoptotic anti-proliferative pathway in the 10AT-Her2 cell population.

### Expression of an I3C-resistant form of elastase in 10AT-Her2 cells disrupts the indole carbinol apoptotic response, and reverses the I3C effects on MDM2–p53, nucleostemin–MDM2 and CD40–TRAF6 protein–protein interactions

**Figure 8:**
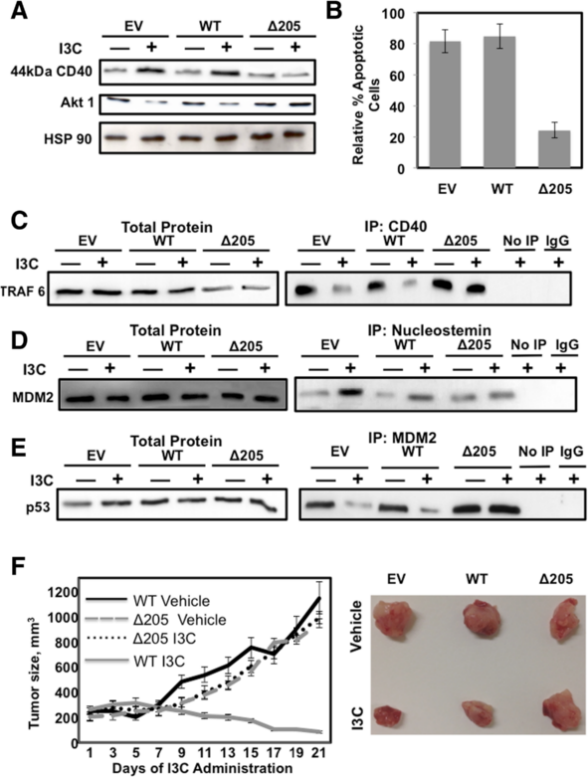
**Elastase-dependent I3C apoptotic effects, regulation of protein–protein interactions, and inhibition of** ***in vivo*** **tumor xenografts. (A)** 10AT-Her2 cells were transfected with either the EV, WT elastase expression vector or the ∆205 truncated elastase expression vector and treated with or without 200 μM I3C for 48 hours. Cell extracts were electrophoretically fractionated and probed by Western blot analysis for CD40, Akt1 and HSP90. **(B)** 10AT-Her2 cells were transfected with EV, WT or ∆205 expression vectors and treated with or without 200 μM I3C for 48 hours. The relative amount of apoptosis was quantified by the ratio of sub-G1 DNA contents as determined by flow cytometry of nuclear DNA stained with propidium iodide. The number of apoptotic cells observed after I3C treatment were normalized to DMSO-vehicle-control-treated cells. **(C, D, E)** 10AT-Her2 cells were transfected with EV, WT or ∆205 expression vectors and treated with or without 200 μM I3C for 48 hours. Total cell extracts were immunoprecipitated with either CD40 **(C)** nucleostemin **(D)** or MDM2 **(E)** antibodies. As controls, immunoprecipitations were carried out with non-immune antibodies (IgG) or not immunoprecipitated (No IP). Cell extracts were electrophoretically fractionated and probed by Western blot analysis using antibodies specific to tumor necrosis factor receptor activator factor-6 (TRAF6) (panel **C**), MDM2 (panel **D**) or p53 (panel **E**). **(F)** 10AT-Her2 cells were stably transfected with EV, WT or ∆205 expression vectors and 300,000 cells were used for xenograft injections of athymic mice. After the formation of detectable palpable tumor xenografts, athymic mice were treated subcutaneously with either 300 mg/kg of I3C or the DMSO vehicle control. Tumor volumes were quantified with a caliper from ten tumor xenografts per condition using two tumor sites per animal. The micrograph insert shows representative tumors excised from 2-week-post-injection animals. DMSO, dimethyl sulfoxide; EV, empty expression vector; I3C, indole-3-carbinol; IgG, immunoglobulin G; IP, immunoprecipitated; MDM2, murine double mutant 2; TRAF6, tumor necrosis factor receptor activator factor-6; WT, wild type.

We previously established that elastase is the biologically relevant indole carbinol target protein in human breast cancer cells, and the non-competitive inhibition of elastase enzymatic activity by I3C triggers a shift from cell survival signaling to apoptotic signaling that is mediated by altered signaling through downstream elastase substrates such as the CD40 member of the tumor necrosis factor receptor gene family [33,34,48]. The indole carbinol inhibition of elastase activity prevents the processing and cleavage of CD40 that results in the stable production of the full-length 44-kDa form of CD40 [34]. Because elastase is the only established direct target protein for I3C, we assessed whether elastase is required for the I3C anti-proliferative signaling in the 10AT-Her2 cell population. To test this possibility, 10AT-Her2 cells were transfected with expression vectors encoding the wild-type (WT) elastase or the Δ205 I3C-resistant form of elastase, which has a carboxyterminal truncation and remains highly enzymatically active but resistant to the inhibitory effects of I3C [48]. Another set of 10AT-Her2 cells was transfected with the EV. Transfected 10AT-Her2 cells were treated with or without I3C for 48 hours. Western blots of total cell extracts demonstrated that in cells transfected with the WT elastase or the EV, the level of the uncleaved 44-kDa form of CD40 is significantly enhanced after I3C treatment (Figure 8A), which is indicative of an inhibition of elastase activity [34]. In 10AT-Her2 cells expressing the I3C-resistant elastase (Δ205), the level of uncleaved CD40 remained low in both indole-carbinol-treated and untreated cells (Figure 8A), which confirms that this truncated elastase remains active under both conditions.

Co-immunoprecipitations revealed that in 10AT-Her2 cells transfected with either the WT elastase or the EV, I3C treatment disrupted the binding of CD40 with one of its immediate downstream effectors, tumor necrosis factor receptor activator factor-6 (TRAF6), which is an E3 ubiquitin ligase that can ubiquitinate Akt-1 [62]. In contrast, in cells expressing the Δ205 I3C-resistant elastase, TRAF6 remained associated with CD40 in the presence or absence of I3C treatment (Figure 8C, right panels). The total levels of TRAF6 remained unaffected by I3C treatment (Figure 8C, left panels). Consistent with the I3C-regulated release of TRAF6 from CD40 in cells expressing endogenous (EV) or exogenous (WT) elastase, the protein levels of Akt-1 decreased in I3C-treated cells, whereas, in cells expressing the I3C-resistant elastase (Δ205), the levels of Akt-1 protein remained unaltered after I3C treatment (Figure 8A, middle panel).

To determine the dependence of elastase in the I3C apoptotic response, 10AT-Her2 cells transfected with the WT elastase gene, the I3C-resistant form of elastase or the EV were treated with or without I3C for 48 hours. Nuclear DNA stained with propidium iodide was analyzed by flow cytometry. The apoptotic response was quantified by the ratio of sub-G1 DNA content in I3C-treated to untreated cells. As shown in Figure 8B, 10AT-Her2 cells expressing the I3C-resistant elastase were mostly resistant to the indole-carbinol-induced apoptosis, whereas cells expressing either endogenous (EV) or exogenous (WT) elastase remained highly sensitive to the anti-proliferative effect of I3C. This result establishes a role for the I3C target protein elastase in mediating the anti-proliferative effects of this natural indole carbinol compound. Co-immunoprecipitation analysis demonstrated that compared to 10AT-Her2 cells expressing endogenous or exogenous WT elastase, expression of the Δ205 I3C-resistant elastase reversed the I3C stimulation of nucleostemin–MDM2 interactions (Figure 8D) and prevented I3C from disrupting MDM2–p53 interactions (Figure 8E). These results directly establish a functional connection between the I3C-dependent regulation of signaling through the I3C target protein elastase and the control of protein–protein interactions directed by the cancer stem/progenitor cell marker protein nucleostemin.

### *In vivo* requirement for elastase in the I3C inhibition of 10AT-Her2 cell-derived tumor xenograft growth

The functional role of elastase in mediating the *in vivo* effects of I3C on tumor xenograft growth was determined by implanting NIH III athymic mice with 300,000 10AT-Her2 cells stably transfected with either the WT or I3C-resistant ∆205 forms of elastase as well as cells transfected with the EV. Tumors were allowed to reach an average volume of 200 mm^3^ before they began to receive subcutaneous injections of I3C or the DMSO vehicle control. Tumor volumes were monitored throughout 21 days. Growth of the tumor xenografts formed with 10AT-Her2 cells transfected with the WT elastase was strongly inhibited in animals injected with I3C compared to animals injected with the vehicle control (Figure 8F, WT I3C vs WT vehicle). Cells expressing the EV were similarly sensitive to I3C (data not shown). In contrast, the tumor xenografts formed from 10AT-Her2 cells stably transfected with the I3C-resistant ∆205 elastase, were completely resistant to I3C and the tumor volumes in I3C-treated and vehicle-control-treated animals were virtually identical (Figure 8F, ∆205 I3C vs ∆205 vehicle). The micrographs in Figure 8 show representative tumor xenografts after 21 days of growth in I3C-treated and vehicle-control-treated animals. In the cells expressing the endogenous or exogenous WT elastase, the tumor xenografts from I3C-treated animals were smaller in size and they appeared less vascularized and displayed pliable texture suggestive of reduced density compared to tumor xenografts from vehicle-control-treated animals. In contrast, the tumor xenografts formed from 10AT-Her2 cells expressing the I3C-resistant ∆205 elastase displayed similar sizes and characteristics in the presence or absence of I3C treatment. Therefore, the I3C regulation of the *in vivo* growth of the 10AT-Her2 cell population highly enriched in cells with cancer stem/progenitor cell-like properties requires expression of the indole-carbinol-sensitive elastase.

## Discussion

Cancer stem/progenitor cell populations have been isolated in clinical samples of breast cancer tissue that characteristically show a CD44^+^/CD24^–^/lin^−^ phenotype [63–67] and also preferentially express other stem cell markers such as nucleostemin and active ALDH-1 [64,68,69]. The ability to evaluate the efficiency of anti-cancer agents in targeting breast cancer stem cells has been limited by the low number of stem cells that can be isolated from tumors and by the loss of viability and/or instability of the stem cell phenotype once the cells are cultured outside the *in vivo* context [63,70–72]. By expressing HER2 in human preneoplastic mammary epithelial cells, forming the 10AT-Her2 cell line, we generated a new human breast cancer cell system in which the overall cell population is highly enriched with cells that stably display breast cancer stem/progenitor cell-like properties. The 10AT-Her2 cell population, but not the EV-transfected 10AT-Neo preneoplastic cells, are CD44^+^/CD24^–/low^/ALDH-1^+^ with high levels of nucleostemin. Analysis of cell surface expression of the CD44 cancer stem/progenitor marker protein by flow cytometry showed that approximately 98% of cells in the 10AT-Her2 cell population express high levels of cell surface CD44, while almost 90% of the cell population contains active ALDH-1. Consistent with the existence of an enriched population of cells with tumor-initiating properties, 10AT-Her2 cells efficiently form tumorspheres in suspension cell cultures and *in vivo* form tumor xenografts with significantly lower numbers of implanted cells compared to well-established highly tumorigenic human breast cancer cell lines.

Based on the expression pattern of nucleostemin, CD44, CD24 and ALDH-1, the stemness character of the SKBR3 human breast cancer population is similar to that of the 10AT-Her2 cell population, whereas MCF-7 and MDA-MB-231 cells express significantly lower levels of nucleostemin and do not produce detectable levels of ALDH-1. These protein expression patterns suggest that the 10AT-Her2 breast cancer cell line does not display abhorrent properties with regard to the production of stem/progenitor cell marker proteins. Several cellular properties of 10AT-Her2 cells suggest that the cell population is more enriched in stem/progenitor cell-like cells compared to the three tested human breast cancer cell lines. For the highly tumorigenic MCF-7 and SKBR3 cells, typically several million cells are required to be implanted into athymic mice in order to detect a high percentage of tumor xenografts. Importantly, in contrast, a near 100% tumor xenograft formation efficiency occurs when only 300,000 10AT-Her2 cells are implanted into athymic mice, and a significant percentage of tumors per inject site can be detected even when as few as 20,000 cells are implanted. Also, the *in vitro* tumorsphere-forming efficiency of the 10AT-Her2 cells is at least 100-fold greater than SKBR3 cells, even though both cell lines express comparable levels of stem/progenitor cell-like marker proteins.

The rationale for expressing exogenous HER2 into preneoplastic mammary epithelial cells to form the 10AT-Her2 cell line is the functional connection between elevated HER2 levels and enhanced cancer stem/progenitor cell populations that can be detected in primary breast tumors and cancer cell lines [13]. Expression of exogenous HER2 in breast cancer cells enhanced the occurrence of side-populations of tumor-initiating cells of the luminal subtype profile [12,13,73,74]. Also, HER2 can be clinically correlated with stem/progenitor cell populations in that patients with HER2^+^ breast cancers treated with the HER2 inhibitor lapatinib show a significant reduction in the number of CD44^+^/CD24^–/low^ cells and a decreased tumorsphere-forming efficiency [12,75,76]. 10AT-Her2 cells and the established SKBR3 breast cancer cell line express generally similar levels of Her2 protein; however, the 10AT-Her2 cells form tumorspheres and tumor xenografts at significantly lower cell numbers compared to SKBR3 cells. Thus, the level of HER2 protein *per se* is not the singular determining factor for the generation of the cancer stem/progenitor cell-like character of our newly developed breast cancer model system. Conceivably a combination of HER2 signaling and the components constituting the preneoplastic phenotype caused the emergence of cells enriched with cancer stem/progenitor cell-like properties in the 10AT-Her2 cell population.

**Figure 9:**
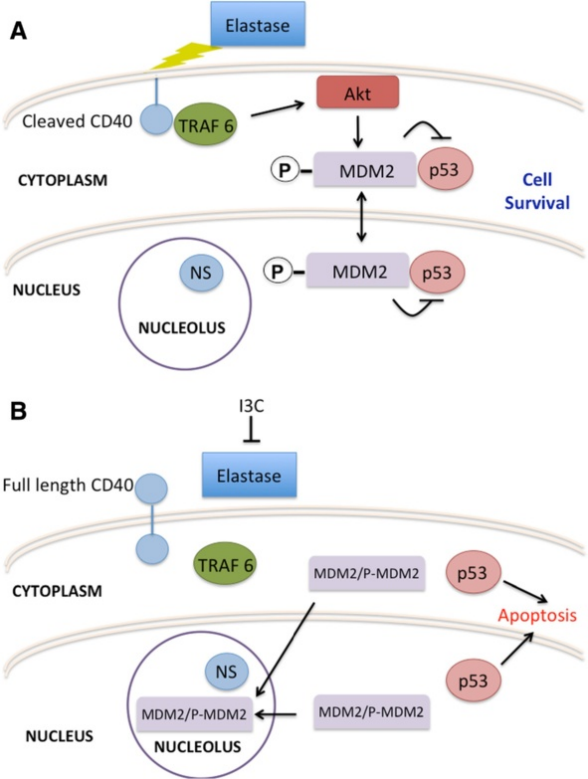
**Proposed model of the elastase-dependent I3C anti-proliferative cascade that targets and requires nucleostemin. (A)** In proliferative cells in the absence of I3C, the elastase cleavage of CD40 triggers signaling through TRAF6 that results in activated Akt phosphorylation of MDM2. As a result, MDM2 interacts with both the nuclear and cytoplasmic forms of p53 and thereby prevents the apoptotic activity of this tumor suppressor protein. Under these proliferative conditions, nucleostemin remains in a nucleolus compartment. **(B)** I3C inhibits the elastase cleavage of CD40 and thereby disrupts the CD40–TRAF6 interaction and inhibits signaling through TRAF6, which results in the loss of MDM2 phosphorylation. The non-phosphorylated MDM2 and phosphorylated MDM2 are sequestered into the nucleolus by their interaction with nucleostemin, which then releases p53 to mediate its apoptotic response. The I3C anti-proliferative cascade is disrupted by expression of an I3C-resistant form of elastase, by the siRNA knockdown of nucleostemin, or by the expression of a dominant negative p53. I3C, indole-3-carbinol; MDM2, murine double mutant 2; NS, nucleostemin; TRAF6, tumor necrosis factor receptor activator factor-6.

We observed that 10AT-Her2 cells are highly sensitive to the anti-proliferative effects of I3C, a natural indole carbinol compound. I3C was shown to trigger a p53-dependent apoptotic response in 10AT-Her2 cells and can disrupt tumorsphere formation in cell suspension cultures as well as inhibit the *in vivo* growth of 10AT-Her2-derived tumor xenografts. Because the 10AT-Her2 cell population expresses relatively high levels of nucleostemin, this system was used to determine whether this breast cancer stem/progenitor marker protein can be potentially targeted by and confer selective responsiveness to I3C. Nucleostemin is a multidomain nucleolus GTPase, which is associated with self-renewal of undifferentiated stem/progenitor cells [60,61]. Co-immunoprecipitations revealed that I3C treatment of 10AT-Her2 cells strongly promoted nucleostemin binding to the MDM2 inhibitor of the p53 tumor suppressor, and thereby sequestered MDM2 into the nucleolus. Although I3C decreased the amount of p-MDM2, this natural indole carbinol compound appears to increase significantly the binding efficiency between phosphorylated MDM2 and nucleostemin. An important consequence of the induced nucleostemin–MDM2 interaction is that I3C treatment prevented MDM2 binding to p53, which we propose allows the p53 tumor suppressor protein to escape the MDM2 inhibition and initiate its cellular apoptotic response (see Figure 9). We also observed the I3C-regulated nucleostemin–MDM2 as well as MDM2–p53 interactions occur in SKBR3 breast cancer cells, but not in either MCF-7 or MDA-MB-231 cells even though all three of these human breast cancer cell lines are sensitive to the anti-proliferative effects of I3C. Conceivably, the attenuated stemness properties of MCF-7 and MDA-MB-231 cells may account for this difference in I3C-regulated protein–protein interaction properties. Consistent with this pathway mediating the anti-proliferative effects of I3C, expression of dominant negative p53 prevented the I3C apoptotic response in 10AT-Her2 cells, whereas knockdown of nucleostemin disrupted the I3C-induced localization of MDM2 into nuclear foci as well as strongly attenuated the apoptotic response. I3C did not alter the protein level of nucleostemin, MDM2 or p53, implying that the key effects are on protein–protein interactions and subcellular localization (see Figure 9).

Knockdown studies of nucleostemin have been controversial as to whether nucleostemin has a cancer promoting or an anti-cancer effect. It appears that the cellular role of nucleostemin is likely to vary in a cell-type-specific manner. Consistent with our observations, the aberrant expression of nucleostemin activates p53 and induces cell cycle arrest via inhibition of MDM2 [60], whereas, depletion of nucleostemin destabilized MDM2 and induced a p53-dependent cell cycle arrest [61]. Relatively little is known about the functional significance of nucleostemin–MDM2 interactions [57,60,61]. Our observations provide the first direct evidence that anti-proliferative signaling by I3C can promote specific interactions of the breast cancer stem/progenitor cell marker protein nucleostemin with the MDM2 inhibitor of p53. It is interesting to note that the presence of nucleostemin *per se* is not sufficient to sequester MDM2 into nucleolus foci because the sequestration of MDM2 is only observed after I3C treatment, even though nucleolus-localized nucleostemin was detected in 10AT-Her2 cells regardless of I3C treatment. We did not observe changes in the total level of p53 protein in several experimental contexts, and our results suggest that the loss of MDM2 accessibility to p53 frees p53 to trigger its apoptotic response. The MDM2 interaction with p53 was partially reversed in I3C-treated cells after knockdown of nucleostemin, suggesting that in the absence of nucleostemin, MDM2 can still interact with p53, although at lower efficiency. It is tempting to speculate that I3C-induced signaling pathways may target nucleostemin and/or MDM2 as a priming mechanism that enhances the nucleostemin–MDM2 protein–protein interaction. Consistent with this possibility, I3C treatment significantly reduced the level of phosphorylated MDM2, which in other systems can influence MDM2–p53 interactions [56]. Phosphorylation of MDM2 at Ser166 by Akt1 is critical in maintaining the MDM2–p53 interaction [77,78]. Our preliminary evidence shows that transfection with constitutively active Akt1 reversed the loss of MDM2 phosphorylation in the presence of I3C, prevented the observed apoptotic response and attenuated the I3C-induced interaction of MDM2 with nucleostemin.

We previously established that elastase is the biologically relevant indole carbinol target protein in breast cancer cells and the noncompetitive inhibition of elastase enzymatic activity by I3C, and other I3C-based derivatives, triggers a shift from cell survival signaling to apoptotic signaling by altering the signaling through downstream elastase substrates, such as the CD40 member of the tumor necrosis factor receptor gene family [34,48]. The elastase cleaved form of CD40 directly interacts with a specific set of downstream effectors (tumor necrosis factor receptor activator factors or TRAFs) to activate cell survival signaling cascades, whereas the uncleaved form of CD40 interacts with a different set of TRAFs to initiate an apoptotic response [34,79,80]. The requirement for elastase in I3C apoptotic signaling in 10AT-Her2 cells was established by expressing the WT elastase or a novel I3C-resistant truncated form of elastase, which remains highly enzymatically active but resistant to the inhibitory effects of I3C [48]. Co-immunoprecipitations showed that in the presence of the WT elastase, I3C treatment released the CD40 binding with TRAF6, which is an E3 ubiquitin ligase responsible for the recruitment of active Akt1 to the plasma membrane [59]. In the presence of the I3C-resistant elastase, CD40 is cleaved in the presence or absence of I3C and TRAF6 remained associated with CD40 under both conditions. Also, in cells expressing the I3C-resistant elastase, treatment with I3C failed to stimulate the MDM2–nucleostemin or inhibit the MDM2–p53 bimolecular interactions, and prevented I3C from triggering its apoptotic response in cell cultures or to inhibit growth of 10AT-Her2 cell-derived tumor xenografts. Therefore, we propose that in 10AT-Her2 cells, which are highly enriched with cells with stem/progenitor cell-like properties, the I3C apoptotic response is triggered by the inhibition elastase and requires the downstream protein–protein interactions of nucleostemin, a stem/progenitor cell marker protein (see Figure 9 diagram).

## Conclusions

An intriguing potential clinical significance of our mechanistic studies is the potential development of I3C-based compounds in new therapeutic strategies that block the emergence and maintenance of stem/progenitor cell populations within breast cancers by inducing a nucleostemin-dependent apoptotic response. Our pre-clinical characterization of the mechanism of action of I3C has shown that the nucleostemin stem/progenitor cell marker protein is essential for the anti-proliferative response of this indole carbinol compound. Given that I3C anti-proliferative signaling requires the presence of WT elastase, which is the only identified direct target protein for I3C, conceivably cancer stem/progenitor cell populations in breast cancers that express both elastase and nucleostemin should be highly sensitive to this indole carbinol compound. In this regard, many advanced-stage breast cancers express high levels of elastase or elastase activity [81,82], and it is tempting to consider that the detection of elastase activity in biopsies could eventually lead to the use of I3C-based compounds for individual patients. Prior to the development of personalized therapies, a critical issue will be to determine whether the cancer stem/progenitor cells that exist in early and/or advanced breast tumors produce high enough levels of elastase and nucleostemin to be sensitive to I3C and its highly potent derivatives. The 10AT-Her2 cell line, which has a stable phenotype and is highly enriched with cells that display breast cancer stem/progenitor cell-like properties, will be used to characterize the precise functional role of signal-regulated alterations in nucleostemin–protein interactions that are part of the anti-proliferative response to I3C, and can be employed to assess the efficiency by which other classes of anti-cancer agents can target specific stem/progenitor cell components in cancer cell populations.

Advanced-stage breast cancers are notoriously difficult to treat but generally possess high levels of WT elastase, the direct target protein that triggers I3C anti-proliferative signaling. Therefore, the broader community-focused significance of our pre-clinical work is the potential to develop low-cost I3C-based compounds as potential cancer stem/progenitor cell-targeted treatment options for women impacted by highly aggressive and metastatic breast cancers.

## Methods

### Expression plasmids and transfections

Human cytomegalovirus CMV-HER2 and CMV-Akt1 expression plasmids were kind gifts from Dr Leonard Bjeldanes (Department of Nutritional Sciences and Toxicology, University of California at Berkeley). The CMV-p53 dominant negative expression vector was a kind gift from Dr Lin He (Department of Molecular and Cell Biology, University of California Berkeley). The WT elastase and I3C-resistant ∆205 elastase expression vectors were as previously described [46]. Transfection of expression vectors was performed using Superfect transfection reagent from QIAGEN (Germantown, MD, USA) per the manufacturer’s recommended protocol.

### Generation of the 10AT-Her2 and 10AT-Neo cell lines and cell culture methods

Preneoplastic MCF-10AT human mammary epithelial cells (obtained from the Barbara Ann Karmanos Cancer Institute, Detroit, MI, USA) were stably transfected with either the human pCMV-HER2 expression vector or with the pCMV-Neo control vector forming 10AT-Her2 cells and 10AT-Neo cells, respectively. Cells were stably selected for 2 months with G418 sulfate, purchased from Cellgro (Manassas, VA, USA). 10AT-Her2 and 10AT-Neo cell lines were cultured in DMEM/F-12, 10% fetal bovine serum, 50 U/ml penicillin/streptomycin (Lonza, Allendale, NJ, USA), 0.02 μg/ml epidermal growth factor (Promega, Madison, WI, USA), 0.05 μ g/ml hydrocortisone, 10 μg/ml insulin and 0.1 μg/ml cholera toxin (Sigma-Aldrich, St Louis, MO, USA). MCF-7 breast cancer cells were cultured in DMEM, 10% fetal bovine serum, 50 U/ml penicillin/streptomycin, 2 mmol/l L-glutamine (Sigma-Aldrich, St Louis, MO, USA), and 10 mg/ml insulin (Sigma-Aldrich, St Louis, MO, USA). SKBR3 breast cancer cells were cultured similarly but without the supplementation of insulin. Cells were grown to subconfluency in a humidified chamber at 37°C containing 5% CO_2_. I3C, DMSO and MG132 were obtained from Sigma-Aldrich. For drug treatments, a 200 mmol/l stock solution of I3C was purchased from Sigma-Aldrich, dissolved in DMSO and then diluted in the ratio 1:1,000 in media before culture plate application. Before each drug treatment, cells were washed in ice-cold PBS, which was obtained from Lonza (Basel, Switzerland).

The 10AT-Her2 cell line, the parental MCF-10AT cells and the SKBR3 breast cancer cell line were validated by short tandem repeat (STR) DNA fingerprinting using DDC Medical Cell Line Authentication Lab Services (Fairfield, OH, USA). The STR profiles were compared to known ATCC fingerprints [83] and to the Cell Line Integrated Molecular Authentication database (CLIMA) version 0.1.200808 [84]. The STR profile of the 10AT-Her2 cell line matched that of its parental MCF-10AT cell line, whereas the SKBR3 cells matched the known DNA fingerprints to that of itself. Therefore, the cell population of the 10AT-Her2 cell line, which displays significant stemness-like character, is not contaminated with any other cell line, such as SKBR3 cells that also display some stemness-like character in the corresponding cell population.

### Tumorsphere formation

Single-cell suspensions of the indicated cell lines were plated on ultra-low attachment plates (purchased from Corning Costar, Corning, NY, USA) in MammoCult Human Medium (Stem Cell Technologies, Vancouver, Canada) and cultured at 37°C, 5% CO_2_. Cells were incubated with or without 200 μM I3C for the indicated times and tumorsphere formation was assessed and quantified visually by phase microscopy.

### Cell proliferation assay

The sensitivity of cells to I3C was examined using the Cell-Counting Kit-8 (Dojindo Molecular Technologies, Inc, Santa Clara, CA, USA) based on the manufacturer’s recommended protocol [53]. Cells were plated at a density of 5,000 to 7,000 cells per well in 24-well plates containing 500 μl of culture medium. After the indicated treatment and incubation times at 37°C, 40 μl CCK-8 reagent was then added to each well, which were then incubated for 2 hours before reading at a wavelength of 450 nm.

### Flow cytometry and ALDEFLUOR assay

The flow cytometry analysis was performed as previously described [49]. To analyze cell surface expression of CD44 and CD24, harvested cells were incubated for 1 hour at 4°C with CD44- or CD24-specific antibodies conjugated to Alexafluor488 secondary antibodies. Cells were suspended in full media and kept on ice pending analysis. The ALDEFLUOR assay of ALDH-1 activity was performed based on the manufacturer’s protocol (Stem Cell Technologies, Vancouver, BC, Canada). Live single cells were gated for analysis using a Beckman-Coulter EPICS XL-MCL flow cytometer (Beckman Coulter, Brea, CA, USA) with a single 488-nm blue laser filtered at 525 BP/slot 1.

### Western blots

After the indicated treatments, Western blots were performed as previously described [51]. Briefly, cells were harvested in ice-cold PBS and lysed using a radioimmunoprecipitation assay buffer (150 mM NaCl, 0.5% deoxycholate, 0.1% NoNidet-p40, 0.1% SDS, 50 mM Tris) containing protease and phosphatase inhibitors (50 g/ml phenylmethylsulfonyl fluoride, 10 g/ml aprotinin, 5 g/ml leupeptin, 0.1 g/ml NaF, 1 mM dithiothreitol, 0.1 mM sodium orthovanadate and 0.1 mM β-glycerol phosphate). After centrifugation, total protein in the lysate was estimated using the protein quantification kit (Bio-Rad, Hercules, CA, USA). Cell lysates were electrophoretically fractionated using SDS-PAGE and transferred to nitrocellulose membranes. The blots were blocked with 5% nonfat dry milk for an hour at room temperature and incubated with primary antibodies overnight at 4°C. Immunoreactive proteins were detected after a 1-hour incubation with horseradish peroxidase conjugated secondary antibodies. Blots were then treated with enhanced chemiluminescence reagents (Estman Kodak, Rochester, NY, USA) for visualization on film. Primary antibodies, nucleostemin, (sc-67012), CD44 (sc-65412), CD-24 (sc-70598), ALDH1 (sc-22588), MDM2 (sc-5304), p53 (sc-6243), and lamin (sc-7293), CD40 (sc-975), TRAF6 (sc-8409) Akt1 (sc-5298) were purchased from Santa Cruz Biotechnology. Actin (AANO1) was obtained from Cytoskeleton (Denver, CO, USA). HER2 (2165), PARP (9542), and phospho-MDM2 (3521 s) were obtained from Cell Signaling Technology, Inc (Danvers, MA, USA). All antibodies were diluted 1:1000 in TBST (0.1 M Tris, 150 mM NaCl, 0.05% Tween 20).

### Co-immunoprecipitation

After the indicated treatments, immunoprecipitations were performed as described previously [85]. Pre-cleared samples were then incubated with 50 μg of specific antibodies as indicated overnight at 4°C. Immunoprecipitated protein was eluted from beads by addition of gel-loading buffer (50 mM Tris–HCl, pH 6.8, 2% SDS, 10% glycerol, 1% β-mercaptoethanol, 12.5 mM ethylenediaminetetraacetic acid (EDTA), 0.02 mM bromophenol blue) and heating the sample at 100°C for 5 min. Samples were analyzed by Western blot. The immunoprecipitation procedure was nearly 100% efficient because the resulting supernates did not contain any of the proteins of interest. If any of the intended immunoprecipitated protein was detected in a given supernatant fraction, that experiment was not used for the study. Also, the “No IP” control in each experiment represents samples that were subjected to the usual immunoprecipitation procedure except that the beads were added without the antibody.

### Transfection of small interfering RNA

Cells were grown and indicated treatments performed on 10-cm tissue culture plates from Nalgene Nuc International (Rochester, NY, USA). Once cells reached 50% confluency, transfection with control or nucleostemin-specific siRNA was performed using HiPerfect transfection reagent based on the manufacturer's protocol (QIAGEN, Germantown, MD, USA).

### Nuclear and cytoplasmic fractionation

10AT-Her2 cells were grown and indicated treatments were performed on 10-cm plates. Once harvested with ice-cold PBS, nuclear extractions were performed using the manufacturer’s guidelines (Thermo Scientific, Rockford, IL, USA). Briefly, cells were vortexed for 15 seconds in cytoplasmic extraction reagent and placed on ice for 1 min. This was repeated twice and then they were centrifuged at 16,000 *g* for 5 min at 4°C. The supernatant of the separated cytoplasm was removed and the pellet was suspended in nuclear extraction reagent and subsequently vortexed for 15 seconds and placed on ice for 10 min. This process was repeated four times and the nuclear sample was then centrifuged at 16,000 *g* for 5 min at 4°C. Samples were analyzed by Western blot analysis.

### Indirect immunofluorescence

Indirect immunofluorescence was performed as previously described [34]. Briefly, cells were grown and indicated treatments performed on two-well chamber slides from the Nalgene Nunc International subsidiary of Thermo Fisher Scientific (Hampton, NH, USA). The cells were fixed with 3.75% formaldehyde in PBS for 15 min at room temperature. After three additional washes with PBS, the plasma membrane was permeabilized with 0.1% Triton-X-100, 10 mM Tris–HCl, pH 7.5, 120 mM sodium chloride, 25 mM potassium chloride, 2 mM ethylene glycol tetra-acetic acid (EGTA), and 2 mM EDTA for 10 min at room temperature. Slides were incubated with 3% bovine serum albumin (Sigma-Aldrich, St Louis, MO, USA) before incubation with primary antibodies. Rabbit anti-nucleostemin antibody and mouse anti-MDM2 antibody was used at a 1:400 dilution. Secondary Alexa 488 anti-rabbit and Texas Red-conjugated phalloidin were each used at 1:400 dilution. The images were acquired and processed by M1/Hamamatsu Orca (Hamamatsu City, Japan) and QImaging MicroPublisher (Surrey, BC, Canada) color cameras.

### *In vivo* tumor xenografts in NIH III nude mice

10AT-Her2 cells (3 × 10^5^), 10AT-Neo cells (3 × 10^6^), WT elastase transfected 10AT-Her2 cells (3 × 10^5^), ∆205 elastase transfected 10AT-Her2 cells (3 × 10^5^), or EV transfected 10AT-Her2 cells (3 × 10^5^) were mixed with an equal volume of Matrigel (BD Biosciences, San Jose, CA, USA) and inoculated subcutaneously into the mammary fat pad region of NIH III mice as previously described [49]. I3C was injected subcutaneously (300 mg/kg body mass) to reduce the potential for formation of I3C condensation products, such as the natural dimer DIM, that normally occur in the acid conditions of the stomach. Stock solutions of I3C (300 mM) were dissolved initially in DMSO and then diluted in appropriate volume of PBS. Mice were also palpated and tumor sizes were measured every other day with calipers. Tumor volumes were calculated using the following equation: *V* = *a* × *b*^2^/2, where *a* is the width and *b* is the length of a tumor [86]. In the indicated experiments, tumor xenografts were excised after the final day of I3C treatments and visualized by light microscopy. Animal experiments were done in accordance with the guidelines of the Office of Laboratory Animal Care (approval ID: OLAC/R116) and under the approval of the University of California, Berkeley Animal Care and Use Committee.

## Abbreviations

ALDH-1: aldehyde dehydrogenase-1
DIM: 3,3'-dimethylindolylmethane
DMEM: Dulbecco's modified Eagle's medium
DMSO: dimethyl sulfoxide
DN: dominant negative
EDTA: ethylenediaminetetraacetic acid
EGF: epidermal growth factor
EV: empty expression vector
HER2: human epidermal growth factor receptor-2
I3C: indole-3-carbinol
IgG: immunoglobulin G
IP: immunoprecipitated
MDM2: murine double mutant 2
Neo: neomycin
NS: nucleostemin
PARP: poly ADP ribose polymerase
PBS: phosphate-buffered saline
Ser166: serine-166
siRNA: small interfering RNA
STR: short tandem repeat
TRAF6: tumor necrosis factor receptor activator factor-6
WT: wild type
